# Preclinical efficacy of dual mTORC1/2 inhibitor AZD8055 in renal cell carcinoma harboring a *TFE3* gene fusion

**DOI:** 10.1186/s12885-019-6096-0

**Published:** 2019-09-13

**Authors:** Eric C. Kauffman, Martin Lang, Soroush Rais-Bahrami, Gopal N. Gupta, Darmood Wei, Youfeng Yang, Carole Sourbier, Ramaprasad Srinivasan

**Affiliations:** 1 Urologic Oncology Branch, Center for Cancer Research, National Cancer Institute, National Institutes of Health, Building 10 - Hatfield CRC, Room 1-5940, Bethesda, MD 20892 USA; 2 Present address: Departments of Urology and Cancer Genetics, Roswell Park Cancer Institute, Buffalo, NY 14263 USA; 3 Present address: Department of Urology and Department of Radiology, University of Alabama at Birmingham School of Medicine, Birmingham, AL 35294 USA; 4 Present address: Department of Urology, Loyola University Medical Center, Chicago, IL 60153 USA; 5 Present address: Office of Biotechnology Products, Office of Pharmaceutical Quality, Center for Drug Evaluation and Research, U.S. Food and Drug Administration, Silver Spring, MD 20993 USA

**Keywords:** TFE3, MITF, Translocation renal cell carcinoma, Fusion gene, mTOR inhibitor, AZD8055

## Abstract

**Background:**

Renal cell carcinomas (RCC) harboring a *TFE3* gene fusion (TfRCC) represent an aggressive subset of kidney tumors. Key signaling pathways of TfRCC are unknown and preclinical in vivo data are lacking. We investigated Akt/mTOR pathway activation and the preclinical efficacy of dual mTORC1/2 versus selective mTORC1 inhibition in TfRCC.

**Methods:**

Levels of phosphorylated Akt/mTOR pathway proteins were compared by immunoblot in TfRCC and clear cell RCC (ccRCC) cell lines. Effects of the mTORC1 inhibitor, sirolimus, and the dual mTORC1/2 inhibitor, AZD8055, on Akt/mTOR activation, cell cycle progression, cell viability and cytotoxicity were compared in TfRCC cells. TfRCC xenograft tumor growth in mice was evaluated after 3-week treatment with oral AZD8055, intraperitoneal sirolimus and respective vehicle controls.

**Results:**

The Akt/mTOR pathway was activated to a similar or greater degree in TfRCC than ccRCC cell lines and persisted partly during growth factor starvation, suggesting constitutive activation. Dual mTORC1/2 inhibition with AZD8055 potently inhibited TfRCC viability (IC50 = 20-50 nM) due at least in part to cell cycle arrest, while benign renal epithelial cells were relatively resistant (IC50 = 400 nM). Maximal viability reduction was greater with AZD8055 than sirolimus (80–90% versus 30–50%), as was the extent of Akt/mTOR pathway inhibition, based on significantly greater suppression of P-Akt (Ser473), P-4EBP1, P-mTOR and HIF1α. In mouse xenograft models, AZD8055 achieved significantly better tumor growth inhibition and prolonged mouse survival compared to sirolimus or vehicle controls.

**Conclusions:**

Akt/mTOR activation is common in TfRCC and a promising therapeutic target. Dual mTORC1/2 inhibition suppresses Akt/mTOR signaling more effectively than selective mTORC1 inhibition and demonstrates in vivo preclinical efficacy against TFE3-fusion renal cell carcinoma.

**Electronic supplementary material:**

The online version of this article (10.1186/s12885-019-6096-0) contains supplementary material, which is available to authorized users.

## Background

Renal cell carcinoma (RCC) consists of distinct subtypes with characteristic histologic features, genetic mutations and clinical behaviors [1]. The RCC subtype harboring an Xp11.2 chromosomal rearrangement (Xp11 Translocation RCC, *TFE3*-fusion RCC, TfRCC) comprises 1–5% of all RCC cases [2–5]. Rearrangements include an inversion or translocation of the *TFE3* gene (Xp11.2), which is a member of the Microphthalmia-associated transcription factor (MiT) family that regulates growth and differentiation [6]. The resulting gene-fusion product links the TFE3 C-terminus with the N-terminus of a fusion partner [e.g. *PRCC* (1q23), *ASPSCR1* (17q25), *SFPQ* (1p34), *NONO* (Xq13) or *CLTC* (17q23)] [6]. Introduction of a constitutively active promoter upstream of the 3′ *TFE3* gene portion is thought to promote carcinogenesis through increased TFE3 C-terminus expression, nuclear localization and transcriptional activity [6]. Characteristic clinical features include common diagnosis in early or mid-adulthood, frequent metastasis at presentation [7] and other atypical risk factors for RCC, including female gender and childhood chemotherapy [3, 7–9]. Defining histologic features include clear and eosinophilic cells, papillary and/or nested architecture, and occasional psammoma bodies [8, 10]. The diagnosis is suggested by young age, tumor histology and nuclear immunoreactivity for the TFE3 C-terminus; however, confirmation of diagnosis requires cytogenetic or molecular evidence of an Xp11 rearrangement or fusion transcript [8, 10, 11].

Effective drug therapies are yet to be identified for TfRCC, and there is no clinical standard for systemic treatment. Prospective drug trials in metastatic TfRCC patients have not been performed due to the lack of known agents with preclinical efficacy. Retrospective studies suggest rapid progression with cytokine therapy and only occasional, partial responses to rapalogs or anti-angiogenesis therapies [2, 12–17]. Mouse models of xenografted TfRCC patient tumor cell lines are established and provide a promising tool for preclinical drug discovery [6].

Novel drug discovery for TfRCC will benefit from identification of key molecular pathways driving this disease [6]. A variety of cellular functions are governed by wild-type TFE3, and the simultaneous dysregulation of these functions might be sufficient to promote carcinogenesis. Key pathways regulated by TfRCC may involve TGFβ, ETS transcription factor, E-cadherin, MET tyrosine kinase, insulin receptor, folliculin, Rb and other cell cycle proteins [6]. Intriguingly, a common connection among these pathways/proteins is the involvement of Akt, a key regulator of cell growth, metabolism and cytoskeletal reorganization. Akt activation is common in many cancers and the target of ongoing clinical trials [18, 19]. We and others have previously described common phosphorylation of Akt in clear cell RCC (ccRCC) tumors and cell lines, including constitutively in the absence of exogenous growth factor stimulation, but similar investigation in TfRCC models is lacking [18–21].

An important downstream target of Akt signaling is the mTOR-containing protein complex, mTORC1, a master regulator of protein synthesis, cellular metabolism and autophagy. Activation of mTORC1 is thought to promote ccRCC carcinogenesis, at least in part, through increased cap-dependent translation of the hypoxia-inducible factor alpha (HIFα) transcript [22]. Selective pharmacologic inhibition of mTORC1 with temsirolimus is approved by the FDA for treatment of high risk metastatic RCC patients and prolongs their survival [23]. However, clinical resistance to mTORC1 inhibition limits its long-term efficacy and may be mediated by several mechanisms, including a feedback loop involving a second mTOR-containing complex, mTORC2, which phosphorylates Akt in response to mTORC1 inhibition [24, 25]. Concomitant targeting of mTORC1 and mTORC2 is an intriguing therapeutic strategy that has been evaluated in several malignancies, including ccRCC, with promising preclinical results [26]. Previous studies have described increased activation of mTORC1 in TfRCC tumors [27, 28], which supports the Akt/mTOR pathway to be a potential pharmacological target for TfRCC [28].

Here we examined Akt/mTOR pathway activation and the preclinical efficacy of dual mTORC1/2 inhibition compared to selective mTORC1 inhibition in TfRCC preclinical in vitro and in vivo models. The results support an important role for Akt/mTOR activation in TfRCC carcinogenesis and identify dual mTORC1/2 inhibition as a systemic therapeutic strategy with in vivo preclinical efficacy against this cancer.

## Methods

### Cell lines and culture

The UOK109, UOK120, UOK124 and UOK146 cell lines had previously been derived from tumors excised from four TfRCC patients who were treated at the National Cancer Institute (NCI, Bethesda, MD), and had been shown to harbor the *NONO-TFE3* or *PRCC-TFE3* gene fusions [29–31]. The UOK111, UOK139 and UOK150 cell lines had been derived from ccRCC tumors excised from RCC patients treated at the NCI and were shown to harbor *VHL* gene mutations [32, 33]. Collection of this material was approved by the Institutional Review Board of the National Cancer Institute and all patients had provided written informed consent. RCC4 was obtained from ECACC General Cell Collection (Salisbury, UK; Cat Nr. 03112702) and the human renal cortical epithelial (HRCE) cell line was obtained from ATCC (Manassas, VA; Cat Nr. PCS-400-011). All cell lines were maintained in vitro in DMEM media supplemented with L-glutamine (4 mM), sodium pyruvate (110 mg/l), glucose (4.5 g/l), and 1X essential amino acids (Gibco, Gaithersburg, MD), with or without 10% fetal bovine serum (Sigma Aldrich, St. Luis, MO). Cell lines were authenticated using short tandem repeat DNA profiling (Genetica DNA Laboratories, Burlington, NC) and confirmed to be mycoplasma-free by LookOut® Mycoplasma qPCR Detection Kit (Sigma Aldrich).

### Immunoblotting

Phosphorylated and total levels of Akt/mTOR pathway proteins were measured by immunoblot in TfRCC and ccRCC cell lines. ccRCC cell lines were used for comparison since we have previously shown that this RCC subtype has frequent constitutive activation of the Akt/mTOR pathway [20]. Akt kinase activation was evaluated by measurement of phosphorylated levels of Akt (Thr308) and Akt (Ser473), the latter also served as a reporter for mTORC2 activation [25], in addition to levels of phosphorylated GSK3β, which is an Akt kinase target. Activation of mTORC1 was assessed by measuring phosphorylated levels of S6 ribosomal protein (Ser240/244) and 4EBP1 (Thr37/46 and Ser65); levels of HIF1α protein, whose translation is suppressed by hypophosphorylated 4EBP1 through its interaction with eIF4E, provided an indirect measure of mTORC1 activity [34]. Levels of phosphorylated mTOR provided additional measures of mTORC1 and mTORC2 activity, where mTOR Ser2448 is activated by S6K1 kinase and reflects amino acid and nutrient status [35] and mTOR Ser2481 autophosphorylation site correlates with intrinsic mTOR catalytic activity [26, 36]. Protein lysates were harvested from cell lines at 60–70% confluency using RIPA buffer (Thermo-Fischer Scientific, Waltham, MA) supplemented with 1 mM PMSF protease inhibitor (Sigma Aldrich). Two-dimensional electrophoretic separation of proteins was performed using 10 μg protein/well in 4–20% gradient polyacrylamide gels (Biorad, Hercules, CA) and transferred onto PVDF membranes (BioRad). Membranes were blocked for 1 h at room temperature in 5% fat-free milk with 0.1% tween, followed by overnight incubation at 4 °C with primary antibody in either fat-free milk and 0.1% tween or TBS with 5% bovine serum albumin and 0.1% tween. Primary antibodies included rabbit anti-P-mTOR (Ser2448), rabbit anti-P-mTOR (Ser2481), rabbit anti-mTOR (total), rabbit anti-P-Akt (Thr308), rabbit anti-P-Akt (Ser473), mouse anti-Akt (total), rabbit anti-P-GSK3β (Ser9), rabbit anti-GSK3β total, rabbit anti-P-S6 (Ser240/244), rabbit anti-S6 (total), rabbit anti-P-4EBP1 (Thr37/46), rabbit anti-P-4EBP1 (Ser65), rabbit anti-4EBP1 (total), rabbit anti-VHL, and mouse anti-β-actin (all from Cell Signaling Technology, Danvers, MA); mouse anti-HIF1α (BD Biosciences, San Jose, CA); and goat anti-TFE3 (Santa Cruz Biotechnology, Santa Cruz, CA). All primary antibodies were incubated at a 1:1000 dilution, with the exception of the anti-VHL and anti-HIF1α, for which a 1:500 dilutions were used. Primary antibody-stained membranes were incubated for 1 h at room temperature with horseradish peroxidase-conjugated secondary antibody, including goat anti-mouse 1:2000 (Cell Signaling Technology), goat anti-rabbit 1:5000 (Cell Signaling Technology) or donkey anti-goat 1:5000 (Santa Cruz Biotechnology). Secondary-antibody stained membranes were developed using a chemiluminescence kit (Pierce, Rockford, IL) followed by radiographic film exposure.

### Drug agents

The dual mTORC1/2 inhibitor, AZD8055 (AstraZeneca, London, UK), was prepared for in vitro assays by dissolution in DMSO to 10 mM (4.65 mg/mL), per manufacturer instructions. The selective mTORC1 inhibitor, sirolimus (Selleckchem, Houston, TX), was prepared for in vitro assays by dissolution in 100% ethanol to 10.9 mM (10 mg/mL). For in vivo assays, AZD8055 was dissolved by sonication in 30% Captisol (CyDex Pharmaceuticals, Lenexa, KS) to a working concentration of 2 mg/ml and pH of 5.0 per manufacturer instructions. For in vivo assays, sirolimus was dissolved in 5% Tween-80 (Sigma Aldrich) and 5% PEG-400 (Hampton Research, Aliso Viejo, CA) to a working concentration of 0.4 mg/ml. Doses of ~ 200 μl drugs were administered to each animal.

### Cell viability assay

Cell viability in vitro was measured using the tetrazolium salt 3-(4,5-dimethylthiazol-2-yl)-2,5- diphenyltetrazolium bromide (MTT, Sigma Aldrich) in a 96-well plate format after 72 h of treatment as previously described [20].

### Cytotoxicity assay

Cell cytotoxicity in vitro was measured with the lactate dehydrogenase (LDH)-based Cytotoxicity Detection Kit (Roche, Indianapolis, IN) using the modified protocol described by Smith et al. [37]. Briefly, 1–5 × 10^3^ cells were plated onto a 96-well plate to achieve approximately 20% cell confluency 1 day after plating, and drug treatment was initiated in pyruvate-free media. Media without cells served as a control for baseline LDH levels in serum (“media control”). After 48 h of treatment, 4 μl Triton X-100 detergent was added to half of the wells for each drug concentration to lyse all live cells (“high controls”). Reaction mixture was made per manufacturer instructions and added to all wells, and absorbance was measured at 490 nm wavelength (Abs_490_). Cytotoxicity for each concentration was calculated as [Abs_490_ (condition) – Abs_490_ (media control)] / [Abs_490_ (condition high control) – Abs_490_ (media control)] [37]. The drug LY294002 was used as a positive control for cytotoxicity induction.

### Cell cycle analysis

Cell cycle analysis was performed following 24-h drug treatment as previously described [38].

### TfRCC mouse xenograft experiments

Animal studies were approved by the NIH Institutional Animal Care and Use Committee (IACUC; Protocol Number: PB-029) and conducted in accordance with US and International regulations for protection of laboratory animals. TfRCC tumor xenografts were generated using the UOK120 and UOK146 cell lines in female immunocompromised athymic Nude mice (*Foxn1*^*nu*^; Jackson Laboratory, Bar Harbor, ME) at 4–6 weeks of age. Mice were housed under specific pathogen free conditions. Briefly, 5 × 10^6^ cells in PBS suspension with 30% (UOK120) or 50% (UOK146) Matrigel (BD Biosciences, Franklin Lakes, NY) were injected subcutaneously into the mouse right flank. When UOK120 (*N* = 34) or UOK146 (*N* = 40) tumors were palpable (volume 0.05–0.20 cm^3^), treatment was initiated with doses of 4 mg/kg sirolimus intraperitoneal (IP) weekly, IP vehicle control weekly (5% Tween-80 and 5% PEG-400), AZD8055 20 mg/kg oral (PO) daily, or PO vehicle control daily (30% Captisol, pH 5.0). 24 UOK120 mice were randomly assigned to receive either AZD8055 (*N* = 12) or PO control (*N* = 12), while 10 UOK120 mice were randomly assigned to receive sirolimus (*N* = 5) or IP control (*N* = 5). 40 UOK146 mice were randomly assigned to receive AZD8055 (*N* = 10), PO control (*N* = 10), sirolimus (*N* = 10), or IP control (*N* = 10). Mouse weights were monitored weekly. Tumor dimensions were measured every 2 days and volume was calculated using the formula: 0.4 *×* (width)^2^*×* (length). Mice were sacrificed by CO2 asphyxiation and cervical dislocation when the longest tumor diameter reached 2 cm per institutional regulations. An additional 8 mice xenografted with UOK120 or UOK146 tumors underwent the same treatments (*N* = 2 mice per treatment) and were sacrificed at 6 h after their first drug dose for analysis of tumor protein. Protein lysates were prepared by mincing tissue and solubilization in RIPA Buffer (Thermo Fisher Scientific). Immunoblotting was performed as described above, with the exception that detection was performed with a Licor Odyssey Imager (LI-COR Biosciences, Lincoln, NE).

Tumor growth of mouse xenografts was compared by calculating linear regressions of growth curves over the treatment period and calculation of *p*-values through a Mann-Whitney test. Survival times were analyzed through a log-rank test and graphed with GraphPad Prism 7.01 (La Jolla, CA).

## Results

### Akt/mTOR pathway activation in TfRCC cells

**Fig. 1:**
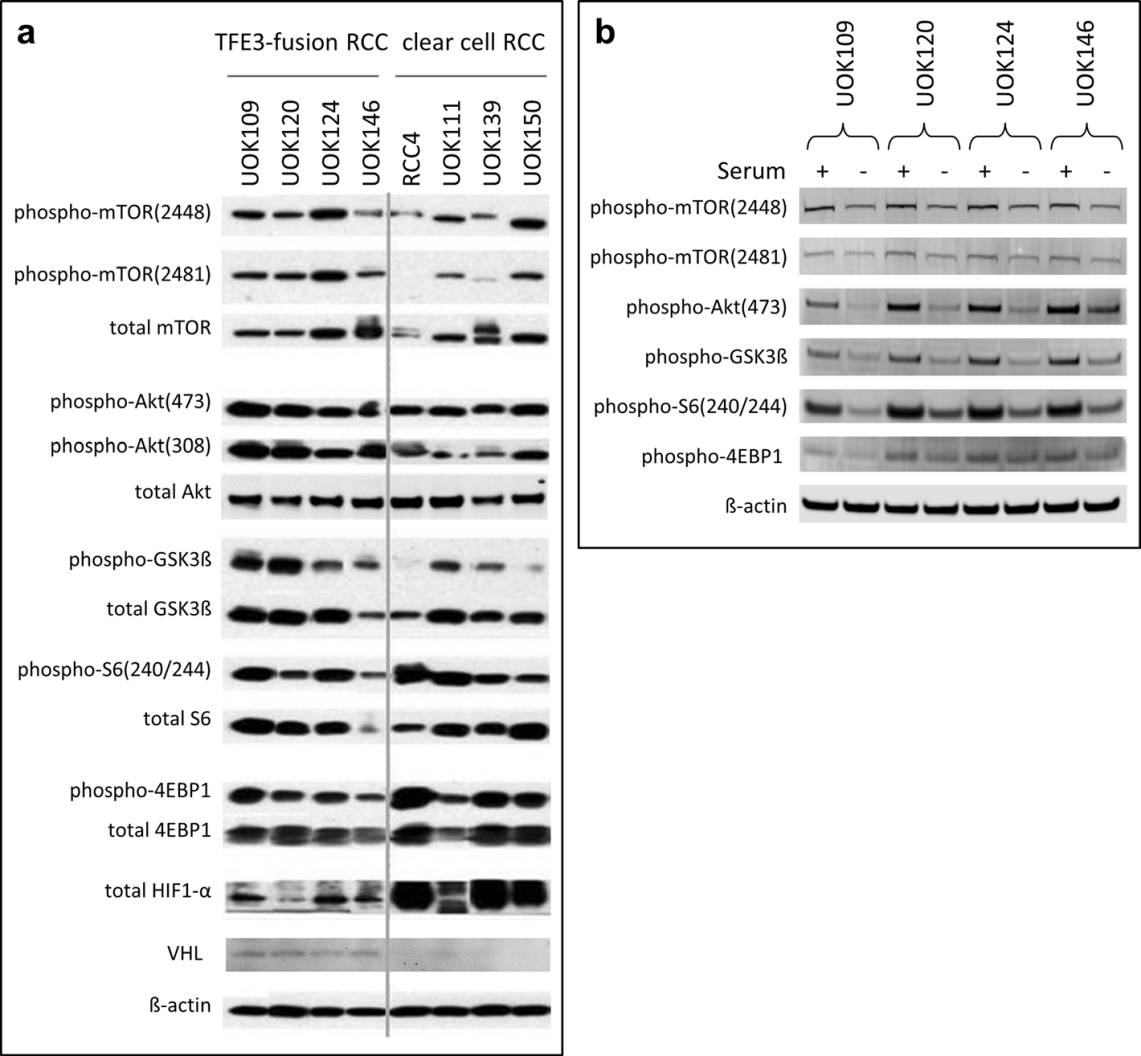
Akt/mTOR pathway member protein expression and activation in TfRCC and ccRCC cell lines. **a** Akt/mTOR pathway member protein expression was determined by Western blot for TfRCC cell lines relative to ccRCC cell lines after 48 h of culture in standard serum-supplemented media. Akt/mTOR pathway activation levels in TfRCC cell lines are comparable to levels in ccRCC cell lines, as shown by similar protein phosphorylation levels of mTOR, Akt, GSK3β, S6 Ribosomal Protein, and 4EBP1. HIF1α expression, a hallmark of ccRCC due to VHL functional loss, is less pronounced in TfRCC than ccRCC cell lines. **b** Akt/mTOR pathway member protein expression was determined by Western blot after serum starvation versus serum stimulation of TfRCC cell lines. Cells were cultured for 18 h in media without serum supplementation followed by culture for 6 h in the presence (+) or absence (−) of 10% serum supplementation. In the absence of serum stimulation, some levels of phosphorylation are preserved in mTOR, Akt, its kinase target protein GSK3β, S6, and 4EBP1, indicating some constitutive activation of mTORC1, mTORC2 and Akt

Akt/mTOR pathway activation was observed in all serum-supplemented TfRCC cell lines (Fig. 1a). Activation of mTORC2 and Akt based on phosphorylated Akt (Ser473) or Akt (Thr308) and phosphorylated GSK3β was more consistently detected in TfRCC than in ccRCC cell lines. Increased levels of phosphorylated S6 ribosomal protein, indicative of mTORC1 activation, was observed in all TfRCC cell lines to an extent comparable with ccRCC cell lines (Fig. 1a). The proportion of total 4EBP1 protein that was phosphorylated was similar between TfRCC and ccRCC cell lines; however, higher levels of both phosphorylated and total 4EBP1 protein were present in ccRCC cell lines. Simultaneous phosphorylation of mTOR at both the Ser2448 and Ser2481 residues was detected in all TfRCC cell lines compared to only a minority of ccRCC cell lines. All TfRCC cell lines expressed VHL and HIF1α protein, although HIF1α levels were much higher in HIF1α(+) ccRCC cell lines compared to any TfRCC cell line, a consequence of posttranslational stabilization due to VHL inactivation in ccRCC [33].

### Constitutive activation of Akt and mTOR in TfRCC cells

To determine whether Akt and mTORC1/2 are constitutively active in TfRCC, levels of phosphorylated mTOR, Akt, S6 and 4EBP1 were measured in the TfRCC cell lines grown in the absence of exogenous serum growth factors as compared to serum stimulation conditions (Fig. 1b). Compared to serum stimulation, phosphorylation levels of all assessed proteins were slightly decreased after serum starvation. However, some level of phosphorylation was maintained for S6 and 4EBP1 even after prolonged serum starvation, indicating that there is some degree of constitutive mTORC1 activation in TfRCC cells. Similarly, persistent phosphorylation after prolonged serum starvation was also observed for Akt at Ser473, supporting some constitutive activation for Akt and mTORC2 in TfRCC cell lines. Phosphorylation of mTOR at Ser2448 and Ser2481 was also largely preserved upon serum starvation. Taken together, these results show some degree of constitutive activation of the Akt/mTORC1/mTORC2 pathway that suggests its importance for TfRCC cell line growth and/or survival.

### TfRCC cell viability in vitro is suppressed more effectively with dual mTORC1/2 inhibition than selective mTORC1 inhibition

**Fig. 2:**
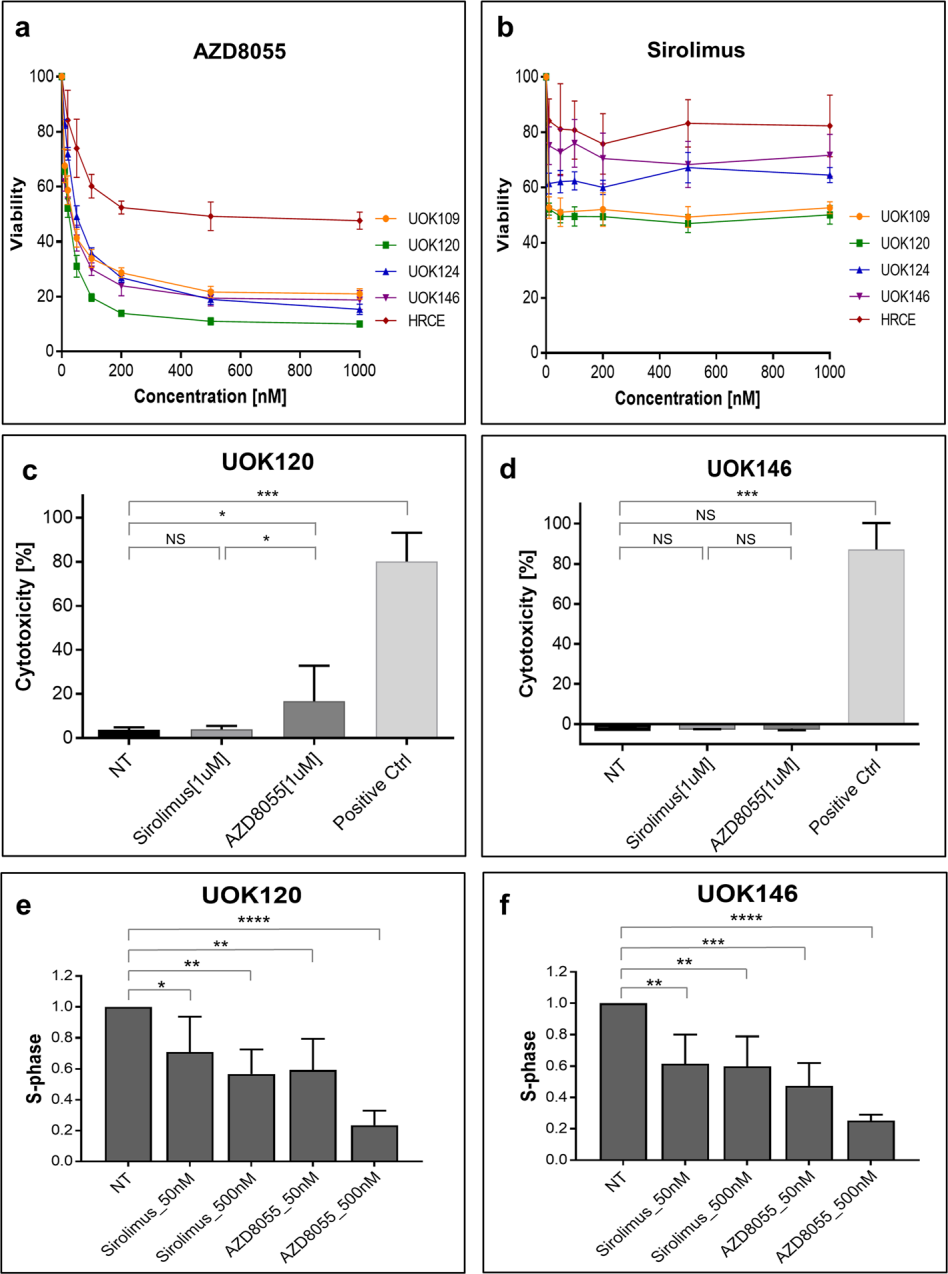
Cell viability, cytotoxicity and cell cycle progression in TfRCC cell lines treated with mTOR inhibitors. **a**, **b** Cell viability, as measured by MTT assay for TfRCC cell lines and the benign renal epithelial cell line HRCE after 72 h of treatment with up to 1000 nM concentrations of the dual mTORC1/2 inhibitor, AZD8055 (**a**), or selective mTORC1 inhibitor, sirolimus (**b**). Viability in TfRCC cells was suppressed by approximately 80–90% with AZD8055 and 30–50% with sirolimus relative to the untreated (0 nM drug) condition. Both drugs inhibited growth to a greater degree in TfRCC cells than in benign renal cells. **c**, **d** Cell cytotoxicity, as measured by LDH release by UOK120 and UOK146 TfRCC cell lines after 48 h of treatment with 1 μM of AZD8055 (**c**) or sirolimus (**d**). Only slight cytotoxicity in UOK120 cells and no cytotoxicity in UOK146 cells was observed after AZD8055 treatment, while sirolimus treatment had no cytotoxic effect. Multi protein inhibitor LY294002 [100 μM] was used as a positive control. **e**, **f** Relative fraction of cells in S-phase of the cell cycle, as measured by BrdU incorporation in UOK120 (**e**) and UOK146 (**f**) cell lines treated for 24 h with low (50 nM) and high (500 nM) concentrations of AZD8055 or sirolimus. Dose-dependent reductions in S-phase in both cell lines with either drug mirror the magnitude of reductions observed in cell viability (**a**, **b**), supporting a predominantly cytostatic mechanism of growth inhibition for both drugs. *p < 0.05; **p < 0.01; ***p < 0.001; NS = non-significant

We performed MTT assays to compare effects of a dual mTORC1/2 inhibitor, AZD8055, and the selective mTORC1 inhibitor, sirolimus, on in vitro cell viability of TfRCC cell lines and the benign renal epithelial cell line, HRCE (Fig. 2). AZD8055 potently suppressed viability in all TfRCC cell lines (IC50 range = 20–50 nM), with maximal viability reduction of approximately 80–90% at 500–1000 nM (Fig. 2a). In contrast, AZD8055 caused relatively little reduction in viability in benign renal cells, with an approximately ten-fold higher IC50 (400 nM) and only 50% maximal viability reduction at 500–1000 nM. An inhibitory effect of sirolimus on viability was observed at low nanomolar concentrations in all cell lines but concentrations above 10 nM had minimal additional effect. Viability suppression of TfRCC cell lines with sirolimus was less effective at higher concentrations compared to AZD8055, achieving only approximately 30–50% maximal reduction at 500–1000 nM. With the exception of UOK120 (IC50 = 50 nM), the IC50 of sirolimus was not reached in TfRCC cell lines at concentrations up to 1000 nM (Fig. 2b). Similar to observations with AZD8055, the inhibitory effect of sirolimus was less in benign renal cell lines (approximately 20% maximal reduction) compared to TfRCC cells.

### Cell cycle arrest contributes to TfRCC growth suppression from dual or selective mTOR inhibition

Because of their ability to generate tumors rapidly in mouse models, the UOK120 and UOK146 cell lines were selected for further in vitro and in vivo studies. First, we examined the mechanism by which AZD8055 and sirolimus inhibited TfRCC cell viability. Activity of LDH released from dying/dead cells was measured in the media of AZ8055- and sirolimus-treated TfRCC cells to determine whether the growth suppression observed in MTT assays was due to cytotoxicity. No significant increase in cytotoxicity was detectable at 1000 nM for sirolimus in the UOK120 and UOK146 cell lines. No cytotoxicity was observed in UOK146 cells and only slight cytotoxicity was observed in UOK120 cells after 1000 nM AZD8055 treatment, despite substantial growth reduction of both cell lines with this dose in MTT assays (Fig. 2c and d). These data suggested that inhibition of cell proliferation rather than induction of cytotoxicity might be the mechanism of TfRCC suppression by AZD8055 and sirolimus. To confirm this hypothesis, cell cycle analysis was performed for the UOK120 and UOK146 cell lines after treatment with either drug. A dose-dependent decrease in S-phase was observed in both cell lines upon treatment with AZD8055, and, to a lower extent, with sirolimus (Fig. 2e and f, Additional file 1: Figure S1). The magnitude of S-phase reduction (~ 30–50% for 500 nM sirolimus, ~ 80% for 500 nM AZD8055) mirrored the magnitude of growth reduction in the MTT assays at similar concentrations. These findings support cell cycle arrest as a primary mechanism by which AZD8055 and sirolimus suppress TfRCC growth.

### Akt/mTOR pathway suppression is more effective with dual mTORC1/2 inhibition than selective mTORC1 inhibition

**Fig. 3:**
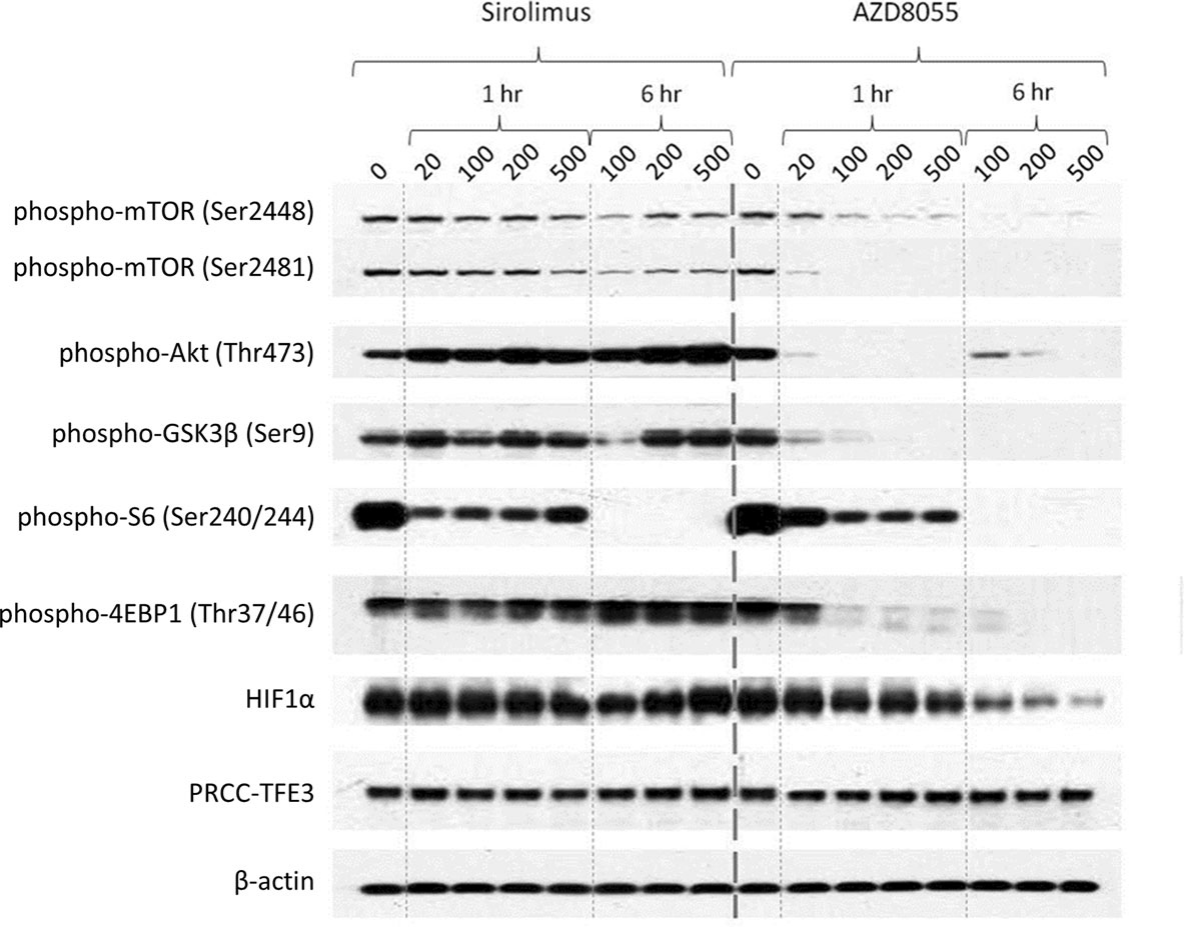
Differential Akt/mTOR pathway suppression in TfRCC cells treated with dual mTORC1/mTORC2 versus selective mTORC1 inhibition. A representative Western blot shows time- and dose-dependent effects of dual mTORC1/2 inhibition with AZD8055 versus selective mTORC1 inhibition with sirolimus in a TfRCC cell line (UOK146). Cells were cultured with 0–500 nM of either drug for 0, 1 and 6 h. Dose- and time-dependent reductions by AZD8055 treatment in levels of phosphorylated S6 or 4EBP1 and Akt (Ser473) confirmed target inhibition of mTORC1 and mTORC2, respectively, with complete suppression of each achieved with 500 nM by 6 h. Similar dose- and time-dependent suppression was observed for other Akt/mTORC pathway members, including phosphorylated GSK3β, phosphorylated mTOR and HIF1α. In contrast, sirolimus achieved complete suppression of phosphorylated S6 by 6 h, but caused time- and dose-dependent increases in other Akt/mTOR pathway members consistent with feedback activation

We next compared effects of AZD8055 and sirolimus treatment on Akt/mTOR pathway activation in TfRCC cells (Fig. 3). Akt/mTOR pathway suppression was more effective with AZD8055 than sirolimus, as demonstrated by more complete downregulation of phosphorylated pathway members (Akt (Ser473), GSK3β, mTOR, 4EBP1) and HIF1α, although S6 phosphorylation was suppressed equally by the two drugs. While AZD8055 suppressed phosphorylated Akt (Ser473), GSK3β and 4EBP1, sirolimus had the opposite effect, increasing each of these phosphorylated proteins in a dose- and time-dependent fashion. Similarly, suppression of HIF1α and phosphorylated mTOR (at either phosphorylation site) by sirolimus was only partial and became progressively less effective with higher sirolimus concentrations. These findings are consistent with feedback activation of Akt/mTOR signaling in response to mTORC1 inhibition, as previously reported [24–26, 39, 40]. In contrast to sirolimus, AZD8055 treatment suppressed phosphorylation of all key Akt/mTOR pathway members to completion in a time- and dose-dependent fashion and achieved nearly 100% reduction in HIF1α protein levels.

### Dual mTORC1/2 inhibition is associated with more effective growth inhibition than selective mTORC1 inhibition in TfRCC mouse xenograft models

**Fig. 4:**
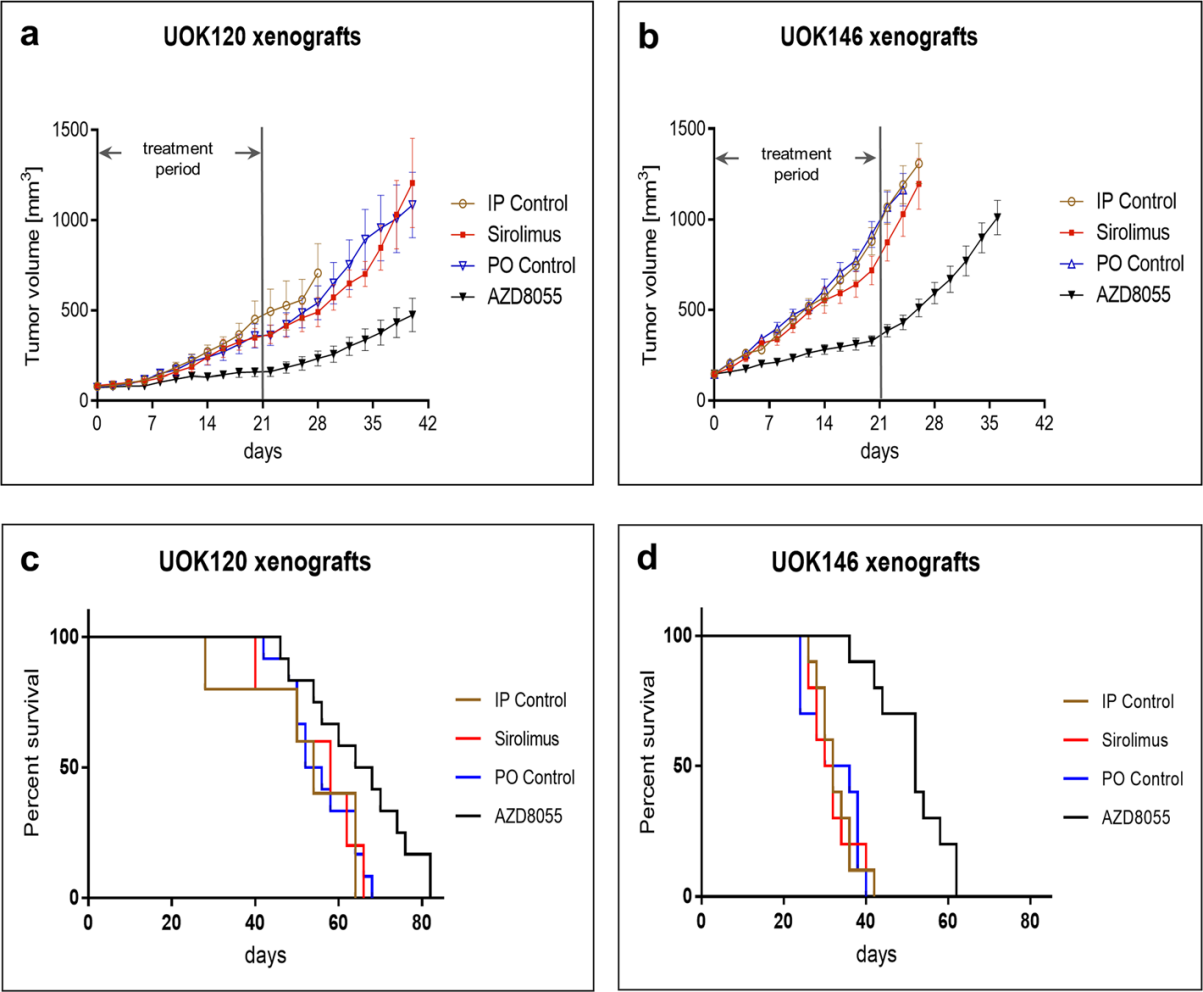
TfRCC tumor growth and mouse survival after treatment with dual mTORC1/mTORC2 versus selective mTORC1 inhibition. Nude mice bearing UOK120 or UOK146 tumor xenografts were treated with oral (PO) AZD8055, PO vehicle control, intraperitoneal (IP) sirolimus or IP vehicle control for a 3-week period. **a**, **b** Tumor growth curves showing average tumor volume over time for each treatment condition in UOK120 (**a**) and UOK146 (**b**) xenograft-bearing mice. AZD8055 significantly reduced tumor size compared to PO control (UOK120: *p* < 0.0001; UOK146: *p* < 0.0001) or sirolimus (UOK120: *p* = 0.004; UOK146: *p* = 0.0003). Growth curves are truncated at the time of the first mouse death for that condition. **c**, **d** Survival curves for xenograft-bearing mice. Sirolimus treatment showed no significant benefit on mouse survival compared to vehicle treated controls, while AZD8055 treatment extended survival compared to the PO control and sirolimus treatments in mice harboring UOK120 (**c**) or UOK146 (**d**) xenografts. Log-rank *p*-values: *p* = 0.021 for AZD8055 vs. PO control (UOK120); *p* = 0.076 for AZD8055 vs. sirolimus (UOK120); *p* = 0.815 for sirolimus vs. IP control (UOK120); *p* < 0.0001 for AZD8055 vs. PO control (UOK146); *p* < 0.0001 for AZD8055 vs. sirolimus (UOK146); *p* = 0.729 for sirolimus vs. IP control (UOK146)

Efficacy of dual mTORC1/2 versus selective mTORC1 inhibition was next evaluated in two mouse xenograft models of TfRCC (UOK120, UOK146). In both models, treatment with AZD8055 resulted in significant inhibition of tumor growth (UOK146: *p* < 0.0001; UOK120: *p* < 0.0001). The mean tumor volume after the 3-week AZD8055 treatment period was reduced by 56% (UOK120) and 64% (UOK146) compared to mice treated with the vehicle control (Fig. 4a and b). However, the suppressive effect of AZD8055 on tumor growth was not maintained following treatment cessation.

In comparison to AZD8055, IP sirolimus resulted in more modest growth inhibition, with tumor volume reductions of approximately 20–25% compared to control mice. In both xenograft models, this tumor volume reduction with sirolimus did not reach statistical significance relative to the corresponding vehicle control (UOK146: *p* = 0.315; UOK120: *p* = 0.691) and was of significantly lower magnitude compared to the reduction achieved with AZD8055 (UOK146: *p* = 0.0003; UOK120: *p* = 0.004). Mouse survival, which was driven by tumor size, was significantly longer in AZD8055-treated mice compared to oral vehicle control-treated mice (UOK146: *p* < 0.0001; UOK120: *p* = 0.021) or sirolimus-treated mice (UOK146: *p* < 0.0001; UOK120: *p* = 0.076) (Fig. 4c and d).

**Fig. 5:**
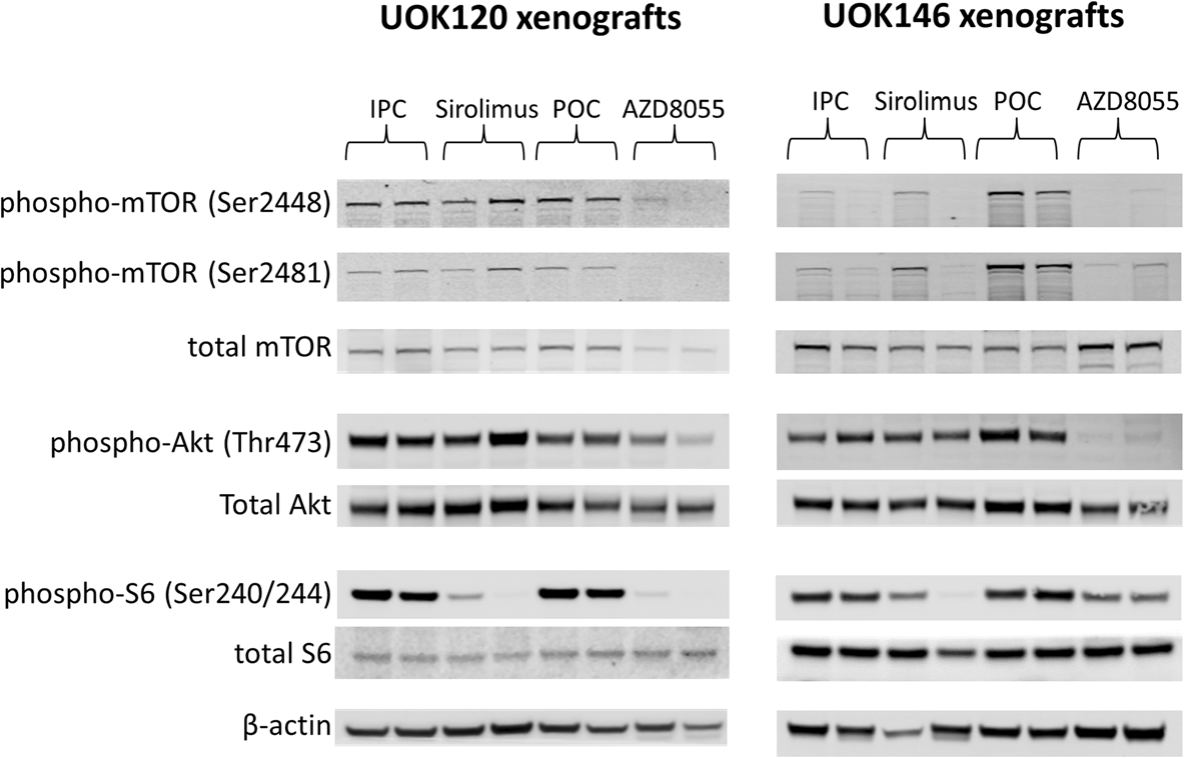
Dual mTORC1/2 inhibitor and selective mTORC1 inhibitor treatments achieve on-target effects in TfRCC xenograft models. Western Blot of UOK120 and UOK146 xenograft tumors 6 h after treatment with a selective mTORC1 inhibitor (sirolimus), a dual mTORC1/2 inhibitor (AZD8055) or respective vehicle controls. Reduction in phosphorylation levels of S6 with sirolimus compared to vehicle control (IPC) confirmed on-target inhibition of mTORC1. Reduction in phosphorylation levels of S6(Ser240/244) and Akt (Thr473) by AZD8055 treatment compared to vehicle control (POC) confirmed on-target inhibition of mTORC1 and mTORC2, respectively. Levels of phosphorylated mTOR were suppressed with AZD8055 but not sirolimus compared to respective controls

Immunoblot analysis of Akt/mTOR pathway members in tumor lysates confirmed on-target effects for both sirolimus and AZD8055 at 6 h after treatment (Fig. 5, Additional file 1: Figure S2). Both drugs achieved complete suppression of S6 phosphorylation indicative of mTORC1 inhibition, while AZD8055 additionally suppressed phosphorylation of Akt (Ser473) indicative of mTORC2 inhibition.

## Discussion

TfRCC is an aggressive RCC subtype with no known effective therapy in the clinical or preclinical setting [2, 12–17]. TfRCC incidence has been historically underestimated because of frequent misdiagnosis as either ccRCC or papillary RCC due to overlapping histologic features, particularly when clinical suspicion for TfRCC (i.e., young age) is otherwise lacking [8]. Retrospective identification of *TFE3-*fusion gene mutations by the TCGA project in several patients diagnosed originally with ccRCC or papillary RCC is consistent with the 1–5% incidence of retrospective identification reported among nephrectomy patients by others [2–5] and may be even higher among metastatic RCC patients. Development of novel therapeutic strategies for TfRCC patients warrants investigation, and identification of key molecular pathways driving TfRCC carcinogenesis is a critical first step.

The current study reveals Akt/mTOR pathway activation in TfRCC cell lines. Akt and mTORC1 pathway activation is common in many human cancers, including ccRCC [18–22] and is mediated by phosphoinositide kinase 1 (PDK-1), the VHL/EGLN suppressive pathway [41], and the mTORC2 complex. mTORC1 activation, as measured by downstream S6 phosphorylation, is reported to be higher in suspected or genetically confirmed TfRCC tumors compared to ccRCC or papillary RCC tumors [27, 28]. We similarly observed high levels of phosphorylated S6 in TfRCC cell lines, comparable to levels in ccRCC cell lines. Levels of Akt activity in TfRCC cell lines generally surpassed those in ccRCC cell lines evaluated and were partly independent of exogenous growth factor stimulation, as previously described for ccRCC [20]. Persistent phosphorylation of mTOR targets in the absence of exogenous growth factor stimulation is consistent with some level of constitutive activation of the mTORC1 and mTORC2 complexes in TfRCC cells. These results suggest that dysregulated Akt and mTOR activation may play an important role in TfRCC carcinogenesis.

To further explore this possibility, we evaluated the efficacy of a dual mTORC1/2 inhibitor, AZD8055, and compared it with a selective mTORC1 inhibitor, sirolimus, in TfRCC cell lines, observing consistently greater growth inhibition with dual mTORC1/2 inhibition. The inhibitory mechanism for both AZD8055 and sirolimus included cell cycle arrest without significant cytotoxicity induction, consistent with the effect of rapalogs reported in other cancer types [42]. Both drugs caused less growth inhibition in benign renal epithelial cells compared to TfRCC cells, indicating a largely cancer-specific effect. Greater growth suppression with AZD8055 than sirolimus in vitro was validated in vivo using two separate mouse xenograft models of TfRCC. These results are consistent with another preclinical study that recently reported PI3K/mTOR pathway dysregulation in TfRCC and suggested that more complete inhibition of this pathway with a dual TORC1/2 and PI3K inhibitor (BEZ-235) results in a greater antiproliferative effect than a selective TORC1 inhibitor [28].

Greater TfRCC suppression with AZD8055 relative to sirolimus is likely due to more complete suppression of the Akt/mTOR pathway. AZD8055- versus sirolimus-treated TfRCC cell lines and mouse xenografts demonstrated clear differences in Akt/mTOR pathway activation. Selective mTORC1 inhibition induced feedback activation of Akt kinase and, consequently, less effective inhibition of downstream S6 phosphorylation, whereas dual mTORC1/2 inhibition suppressed both upstream Akt activation and downstream S6 phosphorylation. Feedback activation of Akt in response to mTORC1 inhibitors is well described in many cancers and may directly mediate clinical resistance in RCC patients [24–26, 39, 40, 43]. Dual mTORC1/2 inhibition blocks this feedback activation and hence provides a promising strategy for overcoming clinical resistance to selective mTORC1 inhibition.

To date, no drug treatment strategy has demonstrated consistent clinical efficacy for metastatic TfRCC patients. Clinical studies are limited by small cohort sizes, retrospective designs, lack of genetic confirmation of *TFE3*-fusion, and heterogeneity in treatment parameters [2, 12–17]. Cytokine therapy is largely ineffective [2, 14–16], and the efficacy of angiogenesis inhibitors has been limited, with progression-free survival typically under 1 year [16, 17]. Similarly, case reports of mTORC1 inhibitors in TfRCC patients suggest rapid progression during treatment [12, 13]. There is hence a clear need for novel therapeutic strategies that broaden the therapeutic target beyond mTORC1. Combinations of mTORC1 and angiogenesis inhibitors have not yet demonstrated clinical benefit over VEGF pathway antagonists alone, and do not address the resistance mechanism of upstream Akt reactivation [44]. The combination of Akt and mTORC1 inhibitors has demonstrated synergistic preclinical efficacy in various cancer types [39, 45]. Dual mTORC1/2 inhibitors such as AZD8055 or Ku0063794 suppress growth of ccRCC cell lines, including those resistant to angiogenesis inhibitors [26, 40]. Although dual mTORC1/2 inhibition with AZD2014 proved inferior to everolimus in metastatic ccRCC patients [46], preclinical studies from our group and others suggest that AZD8055 is superior to rapalogs in ccRCC [40, 47]. The present study extends this prior work to TfRCC, and provides encouraging preclinical rationale for clinical investigation of dual mTORC1/2 inhibition in TfRCC patients [48].

The mechanism underlying constitutive activation of mTOR and Akt in TfRCC warrants future investigation. Activating mutations in the *MTOR* gene have not yet been detected in patient tumors harboring a *TFE3* gene fusion, nor have mutations in *PIK3CA* or *PTEN* [4]. Likewise, genetic characterization of commonly mutated cancer genes in the TfRCC cell lines used in this study did not reveal any pathogenic mutations (unpublished results). Both PI3K and PTEN are implicated as upstream activators of mTORC2 [43]. Given the potential ability of PI3K to activate both mTORC2 and PDK-1, dysregulated PI3K could theoretically explain the high phosphorylation at both Akt (Ser473) and Akt (Thr308) observed in TfRCC. Simultaneous pharmacologic inhibition of PI3K and mTORC1 has demonstrated preclinical efficacy in ccRCC, however dose-limiting toxicity has hindered clinical use [49, 50]. Dual mTORC1/2 inhibition might have lower toxicity owing to its narrower target spectrum, as suggested by a phase I trial of AZD8055 [51]. The MET tyrosine kinase, an upstream activator of Akt, has been proposed to mediate TfRCC carcinogenesis [52], however the putative MET inhibitor, Tivantinib, had no objective responses and poor progression free survival (median 1.9 months) in a small number of RCC patients with a MiT family gene fusion [53]. Such findings warrant reexamination of the importance of MET in TfRCC and are consistent with our prior work showing no significant baseline MET activation in TfRCC cell lines or growth inhibition of these cell lines in response to biologically relevant concentrations of multiple MET-selective inhibitors [6, 54].

## Conclusion

The current study uncovers an important role for the Akt/mTOR signaling axis in TfRCC. Adding to recently published results that suggest therapeutic potential for PI3K/mTOR inhibition in TfRCC [28], our work shows dual mTORC1/2 inhibition suppresses the Akt/mTOR pathway and tumor growth in TfRCC preclinical models more effectively than selective mTORC1 inhibition. These findings provide an encouraging preclinical rationale for the clinical investigation of dual mTORC1/2 inhibitors in TfRCC patients.

## Additional file

Additional file 1: **Figure S1.** Flow cytometry representing suppression of S-phase of cell cycle in TfRCC cells using mTOR inhibitors Cell cycle profile of mTOR inhibitor-treated UOK120 and UOK146 cells measured by flow cytometry and displayed as time-course experiment showing percentage of cells in G2/M-phase, S-phase and G0/G1 phase of the cell cycle at 12 h, 24 h, 48 h and 72 h following drug treatment with 50 nM and 500 nM of Sirolimus and AZD8055 (a and b). Representative scatter plots of total DNA content versus newly synthesized DNA content are shown in c and d. Dose-dependent reduction in the proportion of cells in S-phase is apparent in both cell lines at all time points, with a greater reduction observed using dual mTORC1/2 inhibition (AZD8055) than selective mTORC1 inhibition (sirolimus). An accumulation over time of cells arrested in G0/G1 phase of the cell cycle can be observed. **Figure S2.** Dual mTORC1/2 inhibitor and selective mTORC1 inhibitor treatments achieve on-target effects in TfRCC xenograft models. A quantitative analysis of the changes of phosphorylated protein levels of mTOR pathway proteins in UOK120 and UOK146 xenograft tumors 6 h after treatment with a selective mTORC1 inhibitor (sirolimus), a dual mTORC1/2 inhibitor (AZD8055) or respective vehicle controls (see Fig. 5) is shown as normalized intensity based on β-actin protein levels. (PDF 670 kb)

## Data Availability

All data generated or analyzed during this study are included in this published article and its supplementary information files.

## Abbreviations

ccRCC: Clear cell renal cell carcinoma
MiT: Microphthalmia-associated transcription factor family
RCC: Renal cell carcinomas
TfRCC: TFE3–fusion renal cell carcinoma
