# Supplementary material for: Preclinical efficacy of dual mTORC1/2 inhibitor AZD8055 in renal cell carcinoma harboring a TFE3 gene fusion
Source: BMC Cancer. 2019 Sep 13;19:917. doi: 10.1186/s12885-019-6096-0 (PMC6743205; doi:10.1186/s12885-019-6096-0)
Supplement: Supplementary file 1 — Figure S1. Flow cytometry representing suppression of S-phase of cell cycle in TfRCC cells using mTOR inhibitors Cell cycle profile of mTOR inhibitor-treated UOK120 and UOK146 cells measured by flow cytometry and displayed as time-course experiment showing percentage of cells in G2/M-phase, S-phase and G0/G1 phase of the cell cycle at 12 h, 24 h, 48 h and 72 h following drug treatment with 50 nM and 500 nM of Sirolimus and AZD8055 (a and b). Representative scatter plots of total DNA content versus newly synthesized DNA content are shown in c and d. Dose-dependent reduction in the proportion of cells in S-phase is apparent in both cell lines at all time points, with a greater reduction observed using dual mTORC1/2 inhibition (AZD8055) than selective mTORC1 inhibition (sirolimus). An accumulation over time of cells arrested in G0/G1 phase of the cell cycle can be observed. Figure S2. Dual mTORC1/2 inhibitor and selective mTORC1 inhibitor treatments achieve on-target effects in TfRCC xenograft models. A quantitative analysis of the changes of phosphorylated protein levels of mTOR pathway proteins in UOK120 and UOK146 xenograft tumors 6 h after treatment with a selective mTORC1 inhibitor (sirolimus), a dual mTORC1/2 inhibitor (AZD8055) or respective vehicle controls (see Fig. 5) is shown as normalized intensity based on β-actin protein levels. (PDF 670 kb) [file 12885_2019_6096_MOESM1_ESM.pdf]

Additional File 1

Figure S1: Flow cytometry representing suppression of S-phase of cell cycle in TfRCC cells using mTOR inhibitors.

Cell cycle profile of mTOR inhibitor-treated UOK120 and UOK146 cells measured by flow cytometry and displayed as time-course experiment showing percentage of cells in G2/M-phase, S-phase and G0/G1 phase of the cell cycle at 12 hours, 24 hours, 48 hours and 72 hours following drug treatment with 50nM and 500nM of Sirolimus and AZD8055 (**a** and **b**). Representative scatter plots of total DNA content versus newly synthesized DNA content are shown in **c** and **d**.

Dose-dependent reduction in the proportion of cells in S-phase is apparent in both cell lines at all time points, with a greater reduction observed using dual mTORC1/2 inhibition (AZD8055) than selective mTORC1 inhibition (sirolimus). An accumulation over time of cells arrested in G0/G1 phase of the cell cycle can be observed.

Figure S1a

UOK120 – time course of cell cycle profile

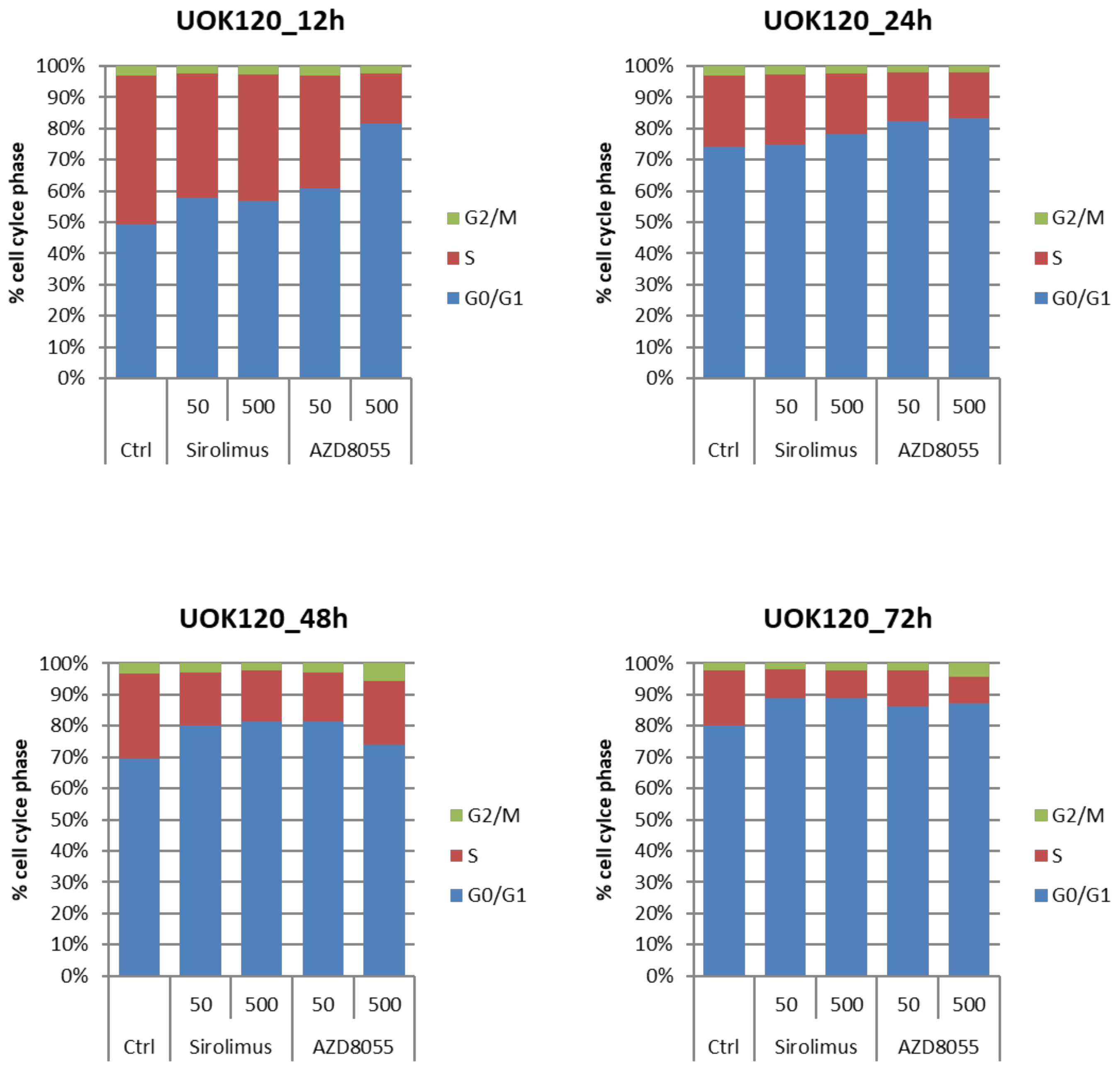

Figure S1b

UOK146 – time course of cell cycle profile

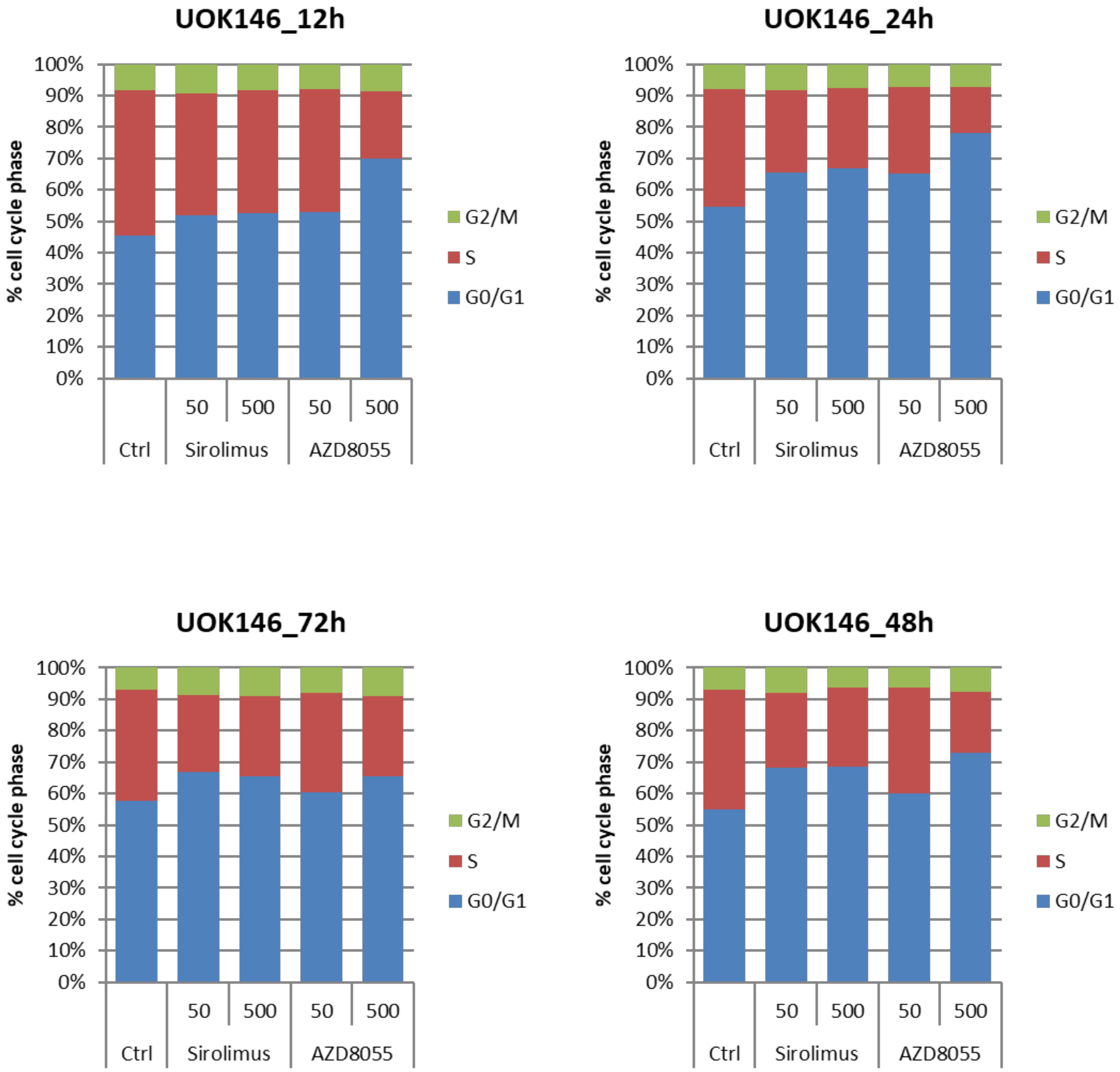

Figure S1c

UOK120 – cell cycle analysis

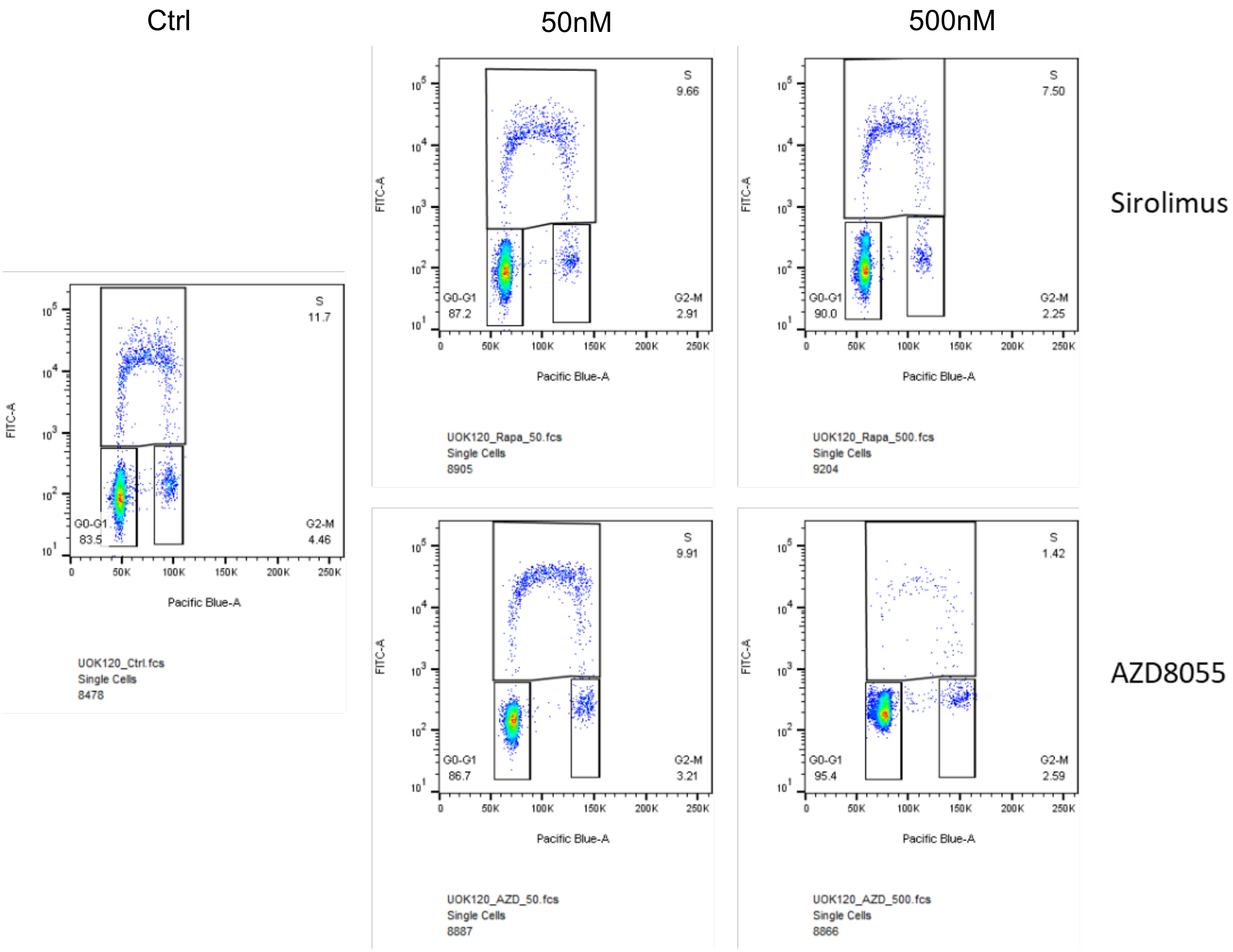

Figure S1d

UOK146 – cell cycle analysis

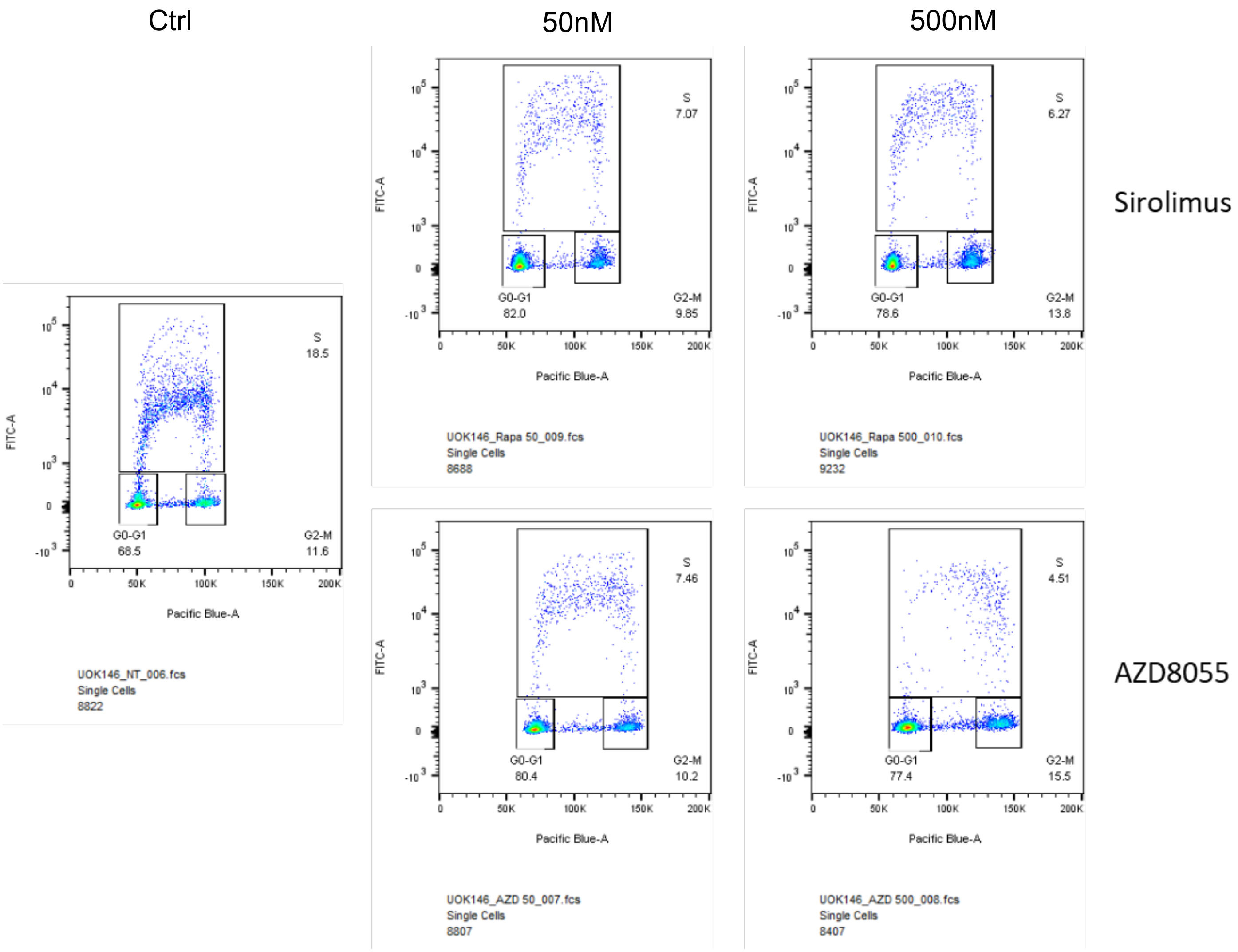

<u>Figure S2</u>: Dual mTORC1/2 inhibitor and selective mTORC1 inhibitor treatments achieve on-target effects in TfRCC xenograft models.

A quantitative analysis of the changes of phosphorylated protein levels of mTOR pathway proteins in UOK120 and UOK146 xenograft tumors 6 hours after treatment with a selective mTORC1 inhibitor (sirolimus), a dual mTORC1/2 inhibitor (AZD8055) or respective vehicle controls (see Fig. 5) is shown as normalized intensity based on β-actin protein levels.

Figure S2

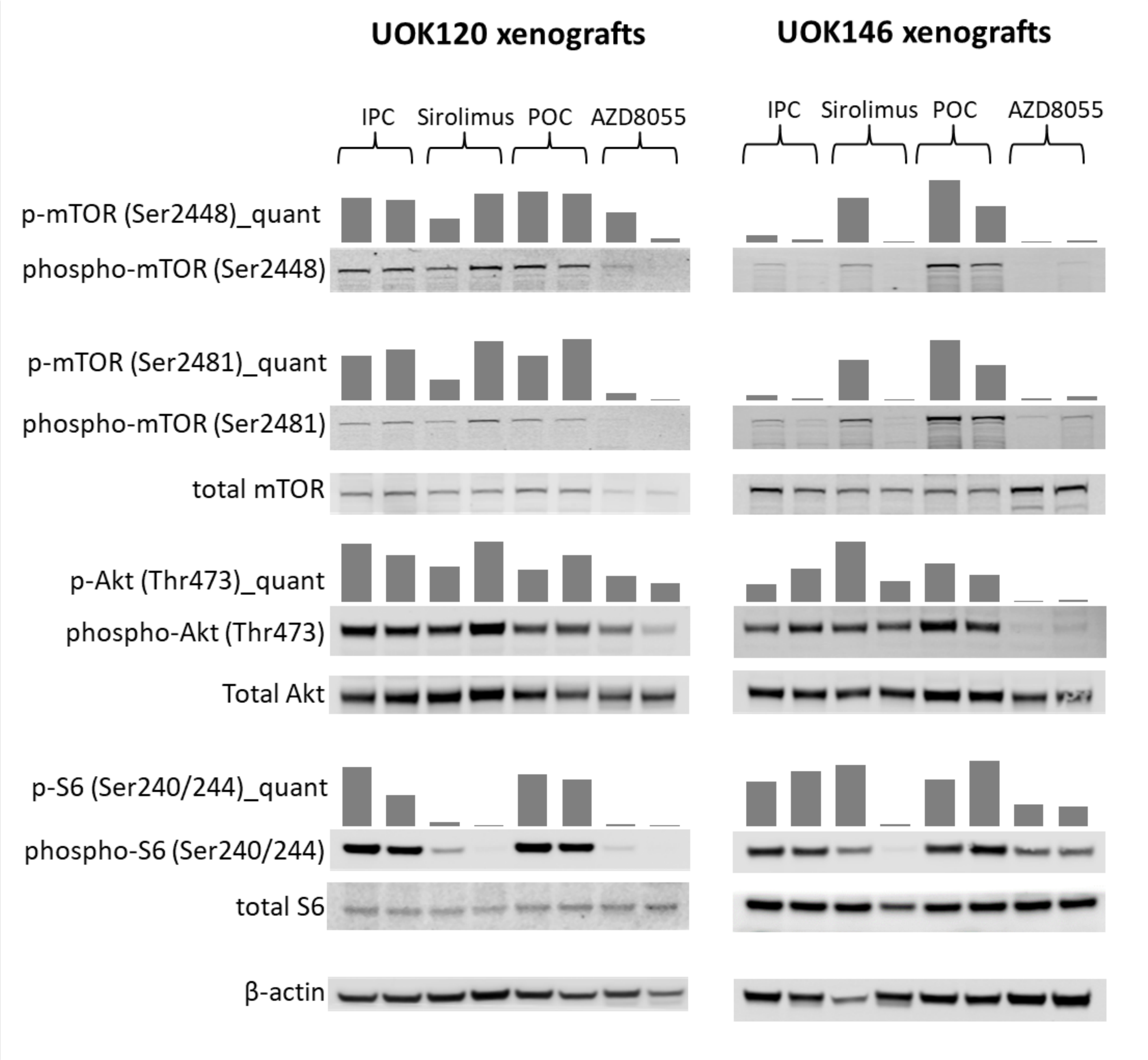

UOK120 xenografts
UOK146 xenografts
IPC
Sirolimus
POC
AZD8055
IPC
Sirolimus
POC
AZD8055
p-mTOR (Ser2448)_quant
phospho-mTOR (Ser2448)
p-mTOR (Ser2481)_quant
phospho-mTOR (Ser2481)
total mTOR
p-Akt (Thr473)_quant
phospho-Akt (Thr473)
Total Akt
p-S6 (Ser240/244)_quant
phospho-S6 (Ser240/244)
total S6
β-actin
